# Kidney disease screening among adults with HIV in Uganda: a missed priority for a high-risk population

**DOI:** 10.1101/2025.08.08.25333274

**Published:** 2025-08-12

**Authors:** Grace Kansiime, Joseph Baruch Baluku, Edwin Nuwagira, Michael Kanyesigye, Paul Stephen Obwoya, Rose Muhindo, Winnie R. Muyindike, Pliers Denis Tusingwire, Henry Mugerwa, Matthew Odera, Monday Busuulwa, Matthew Ssemakadde, Esther C. Atukunda, Francis Bajunirwe, Robert Kalyesubula, Mark J. Siedner

## Abstract

Chronic kidney disease affects 850 million people worldwide, with Sub-Saharan Africa bearing a significant burden. People living with HIV (PWH) are at increased risk due to nephrotoxicity of antiretroviral therapy (ART), in part due to widespread use of tenofovir disoproxil fumarate. In response, Uganda recommends routine kidney disease screening at ART initiation. However, the extent of adherence to these guidelines remains poorly understood.

We extracted clinical data for adults initiating ART between 2017 and 2024 at three large-volume HIV clinics in Uganda. To determine if kidney disease screening rates had increased appropriately over time, we divided the observation period into three eras as per national guidelines: (1) Test and Treat (2017-2019), that recommended screening only PWH and diabetes or hypertension; (2) DTG rollout/COVID-19 (2020-2022); and (3) creatinine-for-all (2023-2024), recommending screening everyone initiating ART. Logistic regression models were fit to identify correlates of renal screening.

Of the 17,485 participants, only 22.4% (3,909/17,485) were screened for kidney disease. Screening was more common at the urban site (54.2%) compared to rural sites (10.0%). At rural sites, screening declined over time and individuals were 83% less likely to be screened in the creatinine-for-all era compared to the baseline era (aOR 0.17, 95% CI: 0.13–0.22) while it increased at urban site (aOR 9.27, 95% CI: 7.37–11.66). Male sex (aOR 1.37, 95% CI: 1.20– 1.57), older age (>45 years), hypertension, and non–TDF-based ART regimens were associated with higher screening odds at rural sites. Diabetes, opportunistic infections, and TDF use were not significantly associated with screening likelihood at any site.

Kidney disease screening at ART initiation remains poor in Uganda, particularly in rural clinics, highlighting critical challenges in translating national guidelines into practice. Future research should focus on understanding multilevel barriers to screening and evaluating strategies to improve guideline uptake.

## Full Text Availability

The license terms selected by the author(s) for this preprint version do not permit archiving in PMC. The full text is available from the preprint server.

